# Parental supply of alcohol and adolescent drinking: a multilevel analysis of nationally representative data

**DOI:** 10.1186/s12889-017-4472-8

**Published:** 2017-06-09

**Authors:** Gary C. K. Chan, Janni Leung, Jason Connor, Wayne Hall, Adrian B. Kelly

**Affiliations:** 1 Centre for Youth Substance Abuse Research, The University of Queensland, QLD, Brisbane, 4072 Australia; 2 Queensland Centre for Mental Health Research, The University of Queensland, Brisbane, Australia; 3 Institute for Health Metrics and Evaluation, University of Washington, Seattle, WA USA

**Keywords:** Adolescent, Alcohol use, Parental supply, Provision

## Abstract

**Background:**

Existing research on parental supply of alcohol analyses the effects of self-reported parental supply on adolescent drinking using individual level data. This study examined the contextual effect of parental supply of alcohol on adolescent alcohol use by examining the association between the prevalence of parental supply in each Australian state and adolescent alcohol use using a multilevel analytic framework.

**Methods:**

Adolescent samples (Age: 12–17) were drawn from the four National Drug Strategy Household Surveys (2004, 2007, 2010 and 2013; *N* = 6803). The prevalence of parental supply of alcohol, defined as the weighted percentage of sample who reported obtaining alcohol from their parents, was estimated in each state and territory across the four surveys. Three multilevel logistic regressions were used to examine the contextual effects of parental supply prevalence on adolescents’ alcohol use in the past 12 months, weekly drinking and heavy drinking.

**Results:**

Overall, adolescents’ rates of past 12 months alcohol use, heavy drinking and weekly drinking between 2004 and 2013 were 40.1, 14.4 and 6.4% respectively. The prevalence of parental supply was significantly associated with past 12 months alcohol use (OR = 1.06, *p* < .001) and heavy drinking (OR = 1.04, *p* < .001) but not with weekly drinking (OR = 1.03, *p* = .189). The results were adjusted for gender, age, socio-economic index for area, place of birth, survey year and prevalence of peer supply.

**Conclusion:**

A high prevalence of parental supply in a region was associated with heavier adolescent drinking, regardless of whether adolescents primarily obtained their alcohol from their own parents.

## Background

Regular adolescent alcohol use, especially heavy use, is associated with many health and behavioural problems, such as violence and injury [1], early sexual debut and risky sexual behavior [2, 3], poor mental health [4], adult alcohol dependence [5] and illicit drug use [6]. Alcohol use is common among adolescents in many western countries. For example, 30% of Grade 8 students in the United States have experimented with alcohol, and 13% reported drinking to intoxication. By Grade 12, these rates have increased to 69 and 54% respectively [7]. In Australia, 46% of 14-year-olds reported alcohol use in the past year, and this rate increased to 81% amongst 17-year-olds [8].

Most Western countries have a legislated minimum age for purchasing alcohol (e.g. 21 years of age in the United States, 18 years of age in Australia and the UK, and 16 years of age in Germany for beer and wine). The high prevalence of adolescent alcohol use despite these age restrictions indicates that adolescents often obtain alcohol from other sources, such as parents and/or peers [9, 10]. Despite the potential harmful effects of alcohol, many parents see its use as an inevitable rite of passage in adolescence and report harm minimization as the rationale for supplying their children with alcohol. Allan and colleagues [11] interviewed Australian parents who gave their children alcohol and found that many believed that by supplying their children with alcohol they could teach them to drink responsibly and provide a safe place to drink, thereby reducing alcohol-related harm in the long term. However, there is little evidence for a protective effect of parental supply of alcohol. Indeed, a review of 22 studies found that parental supply of alcohol was associated with more adolescent alcohol use, heavy episodic drinking and alcohol-related problems [12].

Most of the research on the effect of parental supply has focused on the influence of an individual’s own parents, that is, it has examined the effects of parents providing alcohol to their own children on these children’s alcohol use and alcohol related harm. However, supplying alcohol to one’s own children might also affect alcohol use among other children in their children’s peer group. Little is known about how collective alcohol supply by parents in a region may influence young people’s drinking. Parents who actively supply alcohol to their children might increase alcohol availability in their children’s peer group as their children are likely to share alcohol with their peers. In communities where parental supply is common, adolescents may also have a heightened perception that alcohol is available and that underage drinking is socially endorsed. High perceived availability of alcohol and acceptability of underage drinking are two major community factors that contribute to adolescent alcohol misuse [13, 14].

Parents who supply alcohol to their children also can influence the behavior of other parents in the community. Communities with a high level of parental supply create an environment that encourages all parents to supply their children with alcohol to a level that matches that of their peers [15]. In particular, when parents perceive their friends and peers have positive attitudes towards supplying alcohol to children, they relax their personal objections to adolescent drinking and become much more likely to give their own children alcohol [15]. In this way, the collective action of parents supplying children with alcohol in a region may create a context that increases adolescent alcohol use and its associated risks.

The aim of this study was to examine the contextual effect of parental supply of alcohol on individual adolescents’ alcohol use. We investigated the association between the prevalence of parental supply of alcohol in different regions of Australia and adolescent alcohol use using a multilevel analytic framework. We hypothesized that states with a higher prevalence of parental supply of alcohol would have a higher level of adolescent alcohol use. Given that peers are also a key source of adolescent alcohol, all regression analyses adjusted for the contextual effects of peer supply.

## Method

### Sample

The sample was drawn from tri-annual consecutive National Drug Strategy Household Surveys (NDSHS) conducted in 2004, 2007, 2010 and 2013. The NDSHS is conducted in all Australian States and territories, with an overall sample size of over 20,000 at each survey. For the present study, only data for participants aged 12–17 years were analyzed. The overall sample size of this age group was 6803. The sample size in each year and demographic statistics, including age, gender, and socioeconomic advantage/disadvantage are presented in Table 1. There were no statistically significant differences in age, gender and socio-economic advantage/disadvantage composition across the surveys (*p* > .05). There were significant differences across the surveys in country of birth (*p* < .001). However, the difference was very small (Cramer’s V = 0.07) and the data were weighted to adjust for these imbalances during survey execution. The response rates ranged from 46 to 51% across surveys (Table 1) and these rates were comparable to other Australian surveys [8].

**Table 1:** Sample characteristics

	2004		2007		2010		2013	
	*N*	%	*N*	%	*N*	%	*N*	%
Total sample size	29,445		23,356		26,648		23,855	
Sample size of the age group 12–17	2671	9.07	1455	6.23	1521	5.71	1157	4.85
Male	1250	46.04	725	48.01	738	47.13	577	49.78
Age								
12	386	14.22	205	13.58	232	14.81	151	13.03
13	477	17.57	239	15.83	259	16.54	183	15.79
14	453	16.69	247	16.36	242	15.45	188	16.22
15	463	17.05	256	16.95	275	17.56	197	17.00
16	469	17.27	289	19.14	264	16.86	224	19.33
17	467	17.20	274	18.15	294	18.77	216	18.64
Socioeconomic advantage								
Least advantaged	431	15.87	239	15.86	248	15.84	193	16.65
2nd quintile	518	19.08	275	18.25	314	20.05	211	18.21
3rd quintile	522	19.23	287	19.04	284	18.14	219	18.90
4th quintile	644	23.72	354	23.49	346	22.09	262	22.61
Most advantaged	600	22.10	352	23.36	374	23.88	274	23.64
Australian born	2448	91.65	1283	88.18	1372	90.20	990	85.57
Response Rate		46		49		51		49

### Measures

*Current source of alcohol.* This was assessed by ‘Where do you usually obtain your alcohol now?’ The response options were *friend or acquaintance, brother or sister, parent, spouse or partner, other relative, stole it, purchased it myself from retailer*, *other* and *can’t recall*. Participants were required to mark only one response. Responses from this item were coded into two variables – obtaining alcohol from parents (Yes/ No) and obtaining alcohol from peers (Yes/No).

*Alcohol use.* Three dimensions of alcohol use were assessed – past 12 months alcohol use (Yes/ No), weekly drinking (Yes/ No), and past 12 months heavy drinking (4+ standard drinks in a drinking day; Yes/No). For this age group, the measure of having more than 4 drinks in a drinking day was used as an approximate measure for heavy drinking [16].

*Demographic variables.* Socioeconomic advantage/disadvantage was based on Socioeconomic Indexes for Areas (SEIFA) scores [17]. SEIFA scores are based on population census variables related to disadvantage, such as low income, low educational attainment, unemployment, and dwellings without motor vehicles. Place of birth was coded as “Australia” and “Outside Australia”.

### Procedure

Each NDSHS was approved by the Australian Institute of Health and Welfare Ethics Committee. Access to these survey data by the Centre for Youth Substance Abuse Research was approved by the Australian Social Science Data Archive and by The University of Queensland Human Research Ethics Committee. For each NDSHS, households were randomly selected using a multi-stage stratified design based on statistical local areas [17], with oversampling for small geographical locations.

National surveys were conducted by an independent research company under the direction of the Australian Institute of Health and Welfare (AIHW). Interviewers were located across Australian States and Territories, and underwent training sessions prior to data collection. Data were predominately obtained through a ‘drop and collect’ method across the six surveys. Self-completion questionnaires were delivered and collected to/from households. Householders were provided with a letter from the Director of the AIHW and brochure describing the study, and providing assurances about the confidentiality and anonymity of their participation. Participants were provided with a dedicated free call number managed by the Australian Institute of Health and Welfare to deal with respondent concerns and queries, as well as another dedicated free call number managed by the government-appointed survey contractors. If collection was not possible a pre-paid, pre-addressed envelope was provided and a follow-up reminder telephone call was made. For the 2004 and 2007 surveys, data collection was augmented by face-to-face interviews and/or Computer-Assisted Telephone Interviews. For all methods, the respondent was the household member aged 12 years or above whose birthday was next to occur in the family. Further information on design and methods of the NDSHS can be found elsewhere [18]. To address any potential disparity arising from the survey design or its implementation, and to align the samples with the Australian population, weights were applied to the data based on geographic stratum.

### Analysis

In Australia, there are six states, namely, Queensland (QLD), New South Wales (NSW), Victoria (VIC), South Australia (SA), Tasmania (TAS), Western Australia (WA), and two territories, namely, Northern Territory (NT) and the Australian Capital Territory (ACT). Since the ACT is a small region within NSW and the sample size from ACT was relatively small, samples from ACT and NSW were combined. Point estimates of prevalence of parental supply and peer supply in the seven states/ territories in each survey were calculated. This yielded 28 different point estimates (7 states/territories and 4 surveys) for parental supply and peer supply. Three weighted multilevel logistic regressions were used to examine the contextual effect of prevalence of parental supply and peer supply on heavy drinking, weekly drinking and past 12-month alcohol use. The seven states/territories over four surveys formed 28 Level-2 units in the multilevel analyses. States were chosen as the level 2 unit because (1) this would give a sufficient sample size in each level 2 unit, and (2) the states have independently enacted varying laws and regulations with regard to the secondary supply of alcohol to adolescents; the timing of implementation has also varied. Using states as the level 2 unit allows examination of the contextual effect of parental supply at a state level.

In all three models, the prevalence of parental supply and prevalence of peer supply were entered as Level-2 independent variables; gender, age, socio-economic index for area, place of birth and survey year were entered as Level-1 independent variables. We also adjusted for the individual effect of parental supply in the *Weekly drinking* and *heavy drinking* model. The individual effect of parental supply was not adjusted for in the *Past 12-moth use* model because the time frame of these two variables were different. The item on parental supply was based an item measuring current source of alcohol and the dependent variable was based on item measured alcohol use in past 12 months. Sample weights were rescaled such that the new weights sum to the cluster sample size [19]. The weighted multilevel analyses were performed using the *gllamm* package in STATA [20]. Given that three separate models were run, an alpha level of 0.017 was used to keep the overall family-wise type 1 error rate to be 0.05.

## Results

Overall, the rate of past 12 month alcohol use, heavy drinking and weekly drinking between 2004 and 2013 were 40.1, 14.4 and 6.4% respectively. Table 2 shows the prevalence of parental supply in the 7 states/territories from the 4 surveys. Overall, there was a significant drop in parental supply from 16.2 to 8.1% between 2004 and 2013, *χ* ^2^ (1) = 57.27, *p* < .001. Table 3 shows the results from the three weighted multilevel logistic regressions. Prevalence of parental supply was significantly associated with past 12 months alcohol use (OR = 1.06, 95% CI [1.04, 1.07], *p* < .001) and heavy drinking (OR = 1.04, 95% CI [1.03, 1.05], *p* < .001), but not with weekly drinking (OR = 1.03, 95% CI [0.99, 1.07], *p* = .189). For every 1 % increase in prevalence of parental supply, an adolescent’s odds of past 12 months alcohol use and heavy drinking increased by 6 and 4% respectively. Prevalence of peer supply was only significantly associated with past 12 month alcohol use (OR = 1.04, 95% CI [1.03, 1.06], *p* < .001), and not with heavy drinking (OR = 1.02, 95% CI [0.98, 1.05], *p* = .346) or weekly drinking (OR = 1.03,95% CI [0.98, 1.08], *p* = .323). For every 1 % increase in prevalence of peer supply, an adolescent’s odds of past 12 month alcohol use increased by 4%. In all the analyses, the effect of gender, age, socio-economic index for area, place of birth and survey year were fully adjusted for. In the heavy drinking and weekly drinking model, the individual effect of parental supply was also adjusted for.

**Table 2:** Weighted prevalence of parental supply of alcohol by states/territories between 2004 and 2013

	2004		2007		2010		2013	
	%	95% CI	%	95% CI	%	95% CI	%	95% CI
New South Wales	12.86	(10.21,16.09)	10.00	(7.34,13.50)	4.72	(3.08,7.17)	6.25	(4.01,9.63)
Victoria	16.92	(13.55,20.91)	20.82	(16.49,25.94)	11.78	(8.12,16.81)	12.34	(8.88,16.90)
Queensland	18.39	(15.40,21.82)	18.78	(14.28,24.3)	9.18	(6.39,13.00)	6.27	(3.85,10.05)
Western Australia	18.25	(13.22,24.66)	16.38	(11.34,23.06)	14.01	(8.73,21.72)	4.81	(1.74,12.53)
South Australia	18.78	(13.13,26.13)	20.05	(13.45,28.81)	8.14	(4.03,15.75)	9.99	(5.20,18.35)
Tasmania	23.34	(15.54,33.51)	33.85	(21.05,49.54)	23.67	(13.68,37.77)	14.59	(5.31,34.22)
Northern Territory	14.47	(7.521,26.04)	14.09	(7.17,25.85)	4.16	(1.26,12.86)	16.49	(7.96,31.08)
Overall	16.24	(14.61,18.01)	16.39	(14.41,18.58)	9.03	(7.50,10.84)	8.13	(6.61,9.98)

**Table 3:** Multilevel logistic regression results

	Past 12 months alcohol use		Having 4+ drinks on a day		Weekly drinking	
	OR	95% CI	OR	95% CI	OR	95% CI
Level 1 variables						
Gender (Ref: Male)						
Female	1.12	(0.99, 1.27)	0.85*	(0.74, 0.97)	0.96	(0.72, 1.28)
Age	2.21***	(2.12, 2.30)	2.29***	(2.17, 2.42)	2.32***	(2.13, 2.53)
Socio-economic index for area (Ref: Lowest quintile)						
2nd quintile	1.25	(0.98, 1.60)	1.37	(0.98, 1.93)	1.76**	(1.19, 2.58)
3rd quintile	1.27	(1.00, 1.61)	1.46*	(1.01, 2.10)	1.43*	(1.09, 1.87)
4th quintile	1.16	(0.89, 1.51)	1.24	(0.90, 1.71)	1.27	(0.85, 1.89)
Highest quintile	1.26	(1.02, 1.56)	1.17	(0.86, 1.60)	1.47*	(1.05, 2.06)
Australian born	2.12***	(1.64, 2.76)	2.18**	(1.39, 3.41)	1.56	(0.95, 2.58)
Parental supply of alcohol	^a^		0.87	(0.71, 1.07)	1.04	(0.70, 1.54)
Level 2 variables						
Prevalence of parental supply	1.06***	(1.04, 1.07)	1.04***	(1.03, 1.05)	1.03	(0.99, 1.07)
Prevalence of peer supply	1.04***	(1.03, 1.06)	1.01	(0.98, 1.05)	1.03	(0.97, 1.08)
Survey year (Ref: 2004)						
2007	0.90	(0.79, 1.02)	0.92	(0.82, 1.03)	0.77	(0.54, 1.12)
2010	0.81**	(0.69, 0.94)	1.03	(0.86, 1.23)	0.57*	(0.36, 0.90)
2013	0.65**	(0.51, 0.83)	0.71*	(0.53, 0.96)	0.43**	(0.24, 0.78)

^a^This variable is only included in the model “Having 4+ drinks on a day” and “Weekly drinking” – See analysis section.

***p < .001; **p < .01; *p < .05. The overall model was highly significant for past 12 months alcohol use, *χ* ^2^ (13) = 2133, *p* < .001, having 4+ drinks on a day, *χ* ^2^ (13) = 1115, *p* = < .001, and weekly drinking, *χ* ^2^ (13) =613, *p* = < .001

## Discussion

The present study tested to what extent adolescent alcohol use was associated with prevalence of parental supply in a region. Our results demonstrated that higher prevalence of parental supply in a region was associated with past year alcohol use and heavy drinking among adolescents, after controlling for the prevalence of peer supply. The contextual effect of parental supply on past year alcohol use is similar to peer supply, and the contextual effect of parental supply on heavy drinking was more significant than peer supply. For every one percentage increase in prevalence of parental supply, the odds of adolescent past year drinking and heavy drinking increased by 4 and 6% respectively. Therefore, a 5 % difference in prevalence of parental supply would be translated into 20 and 30% differences in odds of heavy drinking and past year drinking. In particular, the effect of the prevalence of parental supply on heavy drinking was independent of the effect on individuals of supply by their own parents. Our results indicate that adolescents who live in regions where the prevalence of parental supply of alcohol is high are indeed more likely to engage in heavy drinking, regardless of whether his/her own parents provide him/her with alcohol. To our knowledge, the present study was the first study to find any contextual effect of parental supply.

With regards to weekly drinking, although our findings indicated the effect of parental supply prevalence was non-statistically significant, this result should be interpreted with caution. The lower bound of the 95% confidence interval of the odds ratio of parental supply prevalence was just below 1 in the final adjusted model, and the effect was significant in an unadjusted model (a multilevel model included the prevalence of parental supply as the only independent variable, *p* < .001). It is possible that the non-significant results from the final model is due to the effect of parental supply prevalence were mediated through other variables such as peer supply and individual level parental supply. However, given that our dataset is cross-sectional, we were not able to disentangle and draw solid conclusion about the mediation pathway between prevalence of parental supply and other variables. It is also possible that the effect was due to a lack of power. The effect of parental supply prevalence was a contextual effect and entered into the multilevel model as a level 2 variable. Although our overall sample size was large, we only had 28 level 2 units. The small sample size of level 2 units would lead to a limited power to detect an effect. Future research can adopt a longitudinal design with a large sample size to further investigate the association between parental supply prevalence and adolescent frequent drinking.

Overall, our findings are consistent with previous research identifying parental supply as an important risk factor for adolescent alcohol use. Most adolescents first learn about alcohol from their parents and a large proportion obtain their first alcohol from their parents in family settings [9, 10]. When parents supply their children with alcohol, they may send a message to their children that underage drinking is approved and create an environment that encourages drinking [12]. While adolescents usually drink less when they obtain alcohol from parents compared to peers [21], parental supply reduces barriers to alcohol use and promotes progression to unsupervised and harmful drinking patterns (e.g. heavy episodic drinking) [22]. In communities where adolescent alcohol use is sanctioned, adolescents are at higher risk of alcohol use even if they do not obtain any alcohol from their own parents [13].

Our results strengthen the evidence for a focus on parental supply reduction in community-based prevention programs targeting adolescent heavy alcohol use [23]. Community-based prevention approaches typically have a focus on the formation of key local stakeholders, including parent and school groups, and these may yield an economically feasible and potentially sustained impact on a key supply mechanism of adolescent alcohol use. Existing evidence suggests that prevention programs that are coordinated and delivered by communities are effective, but these programs are under-utilized in Australia [24].

Despite its national representativeness, there were some limitations to this study. First, the NDSHS is based on self-report and under-reporting may occur [25]. Second, the NDSHS excludes adolescents without a fixed home and therefore fails to capture some high risk drinkers, such as homeless youth and those in transient accommodation or institutionalized settings. Third, the cross-sectional nature of the survey precludes conclusion about causation. Fourth, participants in our study were grouped into relatively large geographical regions (i.e. state/territory) as level 2 units for the multilevel analysis due to small sample size in each survey. Smaller units such as neighborhood or community groups might provide better estimates for the contextual effect. Therefore, the results we found were conservative estimates as the effects were averaged across all neighborhoods and communities within a state. The actual effect might be larger in smaller locations with more homogeneity in parental supply. Fifth, the item on alcohol source asked the participants to indicate their usual source and participants were prompted to identify one source only. Therefore, the prevalence of parental supply was likely to be an underestimate because participants who indicated they usually sourced alcohol from their peers might also occasionally source their alcohol from parents.

## Conclusions

There is evidence for a contextual effect of parental supply on adolescent drinking. The contextual effect of parental supply on past year alcohol use is similar to peer supply, and the contextual effect of parental supply on heavy drinking is more significant than peer supply. Adolescents living in states where the prevalence of parental supply is high are at increased risk of heavy drinking, regardless of whether their own parents provide them with alcohol.

## Abbreviations

NDSHS: National Drug Strategy Household Survey
